# Recognition of Homeopaths and the Code of Medical Ethics

**Published:** 1898-09

**Authors:** 


### EDITORIAL DEPARTMENT.

F. E. DANIEL, M. D., Editor.
S. E. HUDSON, M. D., Managing Editor.
A. J. SMITH. M. D., Galveston, Associate Editor.

Published Monthly at Austin, Texas, by Drs. Daniel and Hudson. Subscription price $1.00 a year in advance.

Eastern Representatives: Messrs. Monihan & Fairchild, St. Paul Building, 220 Broadway, New York City.

Official organ of the West Texas Medical Association, the Houston District Medical Association, the Austin District Medical Society, the Brazos Valley Medical Association, the Galveston County Medical Society, and several others.

### RECOGNITION OF HOMEOPATHS AND THE CODE OF MEDICAL ETHICS.

We publish in this issue an address to the medical profession of Texas, by the Committee on Legislation appointed by the Texas State Medical Association, and bespeak for it a careful examination.

It is an able address. It sets forth, in a clear and forcible manner, the needs for medical legislation in this State—a subject almost threadbare with repetition.

But the ends proposed to be accomplished, it seems to me, do not "justify the means." I cannot take any other view of the subject than that it is a serious mistake,—one which in the fullness of time will be recognized and repented of; that it is in contravention of the spirit and the letter of the code of medical ethics—the *Magna Charta* of the great medical profession of America,—and is a complete reversal—a "going back on" the history, traditions and record of American medicine.

The gentlemen who are urging the adoption by the medical profession of Texas of the draft of a bill extending recognition to, and fellowship with homeopaths, are perfectly familiar with the code; it is unnecessary to quote it. And it does seem to me most remarkable that they cannot or will not look at this grave question from the standpoint of professional ethics; but rather, sink ethics in what I believe to be a mistaken notion of expediency. These gentlemen speak for the Texas State Medical Association, which body, as I have before pointed out, does not embrace exceeding six per cent of the rank and file of the medical profession of Texas; the appeal is made to the other ninety-four per cent.

In some quarters, I am credited with defeating the bill proposed by the State Association and presented to the last legislature. It will be remembered that that bill provided for a State Board of Medical Examiners, to be composed of six physicians (representing the medical profession of Texas, estimated at over four thousand), *four homeopaths* (representing about one hundred, all told, in the State), and two Eclectics (number in Texas not known, but very small). In this the gentlemen do me too much honor; there were other causes operative to defeat the measure besides my well-known opposition; but I own up to my share in it, and would be proud to "confess the soft impeachment." The opposition of one member to the united wishes of the State Association in the matter of thus extending recognition to homeopaths as a "school of medicine" and proposing to fraternize with them, would be as absurd perhaps as was the conduct of the one juryman, who said the other eleven were most obstinate, or words to that effect, were it not that I honestly believe that the majority of the medical men of Texas —the ninety-four per cent outside of the Association—are of my way of thinking, and are opposed to any such measure. This may perhaps account for the persistency with which they hold aloof from joining that body.

Now, I cheerfully accord to my distinguished colleagues of the Texas State Medical Association, perfect honesty and sincerity of convictions in the belief that they are doing, or trying to do the best thing for the profession and for the people. But, as I said before, I cannot bring myself to see it from their standpoint. There are certain inconsistencies which I cannot reconcile. I cannot reconcile the recognition of and proposed fraternity with homeopaths with even the most liberal construction of the code. It is distinctly forbidden by the code.

I cannot reconcile it with the action of the Association at Tyler, in April, 1897, on which occasion, after the adoption of the bill mentioned above, there was unanimously adopted a resolution denouncing consultation with homeopaths as a violation of the code; and a threat was embodied and recorded "*to expel any member who should so lower the dignity of his profession as to consult with a homeopath.*"

I cannot reconcile it with the record made by the Association at Galveston, in 1877, when the conduct of Dr. Ross, in associating with a homeopath on a board of examiners, was bitterly denounced, and the old doctor was virtually driven from the body, though not expelled formally.

It is, in my mind, a serious question whether a State Association would not justly and properly be refused representation in the American Medical Association upon a showing that its members collectively—while denouncing and repudiating homeopaths, individually—had asked for a bill recognizing homeopaths, and proposing to affiliate with them as medical examiners. It is an issue that I shall (D. V.) take upon myself to make before the Judicial Council of that body at the next meeting, and forever, I hope, set the matter at rest. At Milwaukee, in 1884, the American Medical Association refused to seat delegates from the New York Medical Society, a body which had repudiated the code, and was known as the "New Code men," or "No Code men."

Meantime the following interrogatories are respectfully submitted for consideration. I would be glad to receive replies to them for publication. Doubtless the friends of the bill can say something in justification of the proposed action further than that the "Constitution of the State recognizes homeopathy as a 'school of medicine.'" I will say here, parenthetically: the constitution of the United States recognizes property in black slaves, or did, at the time the United States, by force of arms, abolished slavery.

Do any of the gentlemen who favor recognition of homeopaths as a "school of medicine," and favor exempting them from examination in rational therapeutics and practice, know a single homeopath who sticks to his *similia similibus curantur* and his little pills, and who does *not* use the remedies prescribed by the United States Pharmacopœa? use the hypodermic syringe, and other means and methods of treatment, diametrically the opposite of his professed principles? I do not. If, then, they profess to be homeopaths; advertise themselves as such, accept calls—get practice—as such, and then abandon all pretense of the alleged principles of homeopathy and attempt the practice of rational medicine, a science to which they do not even pretend to be educated, what are they but frauds and quacks? and in the name of all that is honest and consistent, upon what ground can they claim exemption from examination in all the branches of medicine, or claim the right—proposed to be accorded to them—of being examined by a homeopathic board and in homeopathy only? Homeopathy, pure and simple—if there be such thing—is no part of medicine. When an alleged homeopath attempts to practice medicine, why should any distinctions be made in his favor? Why should he not be required to conform to the standard fixed for others who engage in the practice of medicine?

In all kindness I will say, I am no enemy to the State Association. In years past they have honored me. It is because I love the medical profession, and honor its traditions and history—a profession to which, early wedded, I have given nearly thirty-six years of my life and my best endeavors—that I oppose what seems to me to be a total surrender of principle, a scouting of the letter and spirit of our code of ethics. Better far, abandon all attempts to secure medical legislation to protect the people from quackery, than to ally ourselves with a handful of the most notorious and blatant quacks that disgrace the very name of medicine. It is because I am a friend of the Texas State Medical Association,—because, for years, the TEXAS MEDICAL JOURNAL has borne aloft the banner of ethical medicine,—advocated higher medical education, and denounced homeopathy and all other forms of quackery, that I still urge my views, and protest against what I do honestly believe to be a sad mistake on the part of the framers of the bill in question; a mistaken notion of expediency. The occasion does not demand the sacrifice it is proposed to make.

Notwithstanding what I have said above, for personal reasons and in deference to the wishes of friends who desire the passage of the bill in question, I will not oppose its passage should it be presented to the legislature.

